# Brain and Fingers

**Published:** 1891-04

**Authors:** 


### Brain and Fingers.

In discussing educational advantages that the future student of dentistry should be required to attain in the course of study.

Dr. Geo. H. Wilson, of Painesville, said at the Ohio State Dental Meeting. "We should have brain in our fingers, and the student should be taught manipulation as well as any other part of their studies." This is only too true for we often see a clumsy looking hand made expert by education of its muscles, whereas a shapely hand is often worthless, so to speak, from its lack of usage. Then too, there is much truth in the saying "brain in the fingers," which is perhaps more than figurative language. Let us see. Nerve fibers are known to be the medium through which impulses are conveyed to nerve centre or conducted to their terminal filaments dependent upon the stimulus to create such action. In the manipulation of the fingers, in performing operations two factors are at once called into action and each facter involves other factors. Consequent upon the operation to be performed, the first factor may be that of *expression*, or, *impression*. In simple manipulation the eye perceives the condition and the brain telegraphs the mode of expression. When the eye must be assisted by the sense of touch we then have to receive impressions from the point of contact before expression is given to the mind's desire, which is still another factor in the case. Co-ordinate action of all these factors make up what we call manipulation. It is the education of these out-posts, so to speak, that produces skilled action. The mind might perceive what would be necessary to do, but the "outposts" are unable to perform intelligently without education. Inquiry concerning the names of these efficient soldiers reveal the tactile corpuscles of meissner which are found in the skin, and the terminal nerve fibers in the muscles of the hand and arm. These are the anatomical factors that we should cultivate as they in a large measure are the distributions of brain constituents.

"WOULD any shock at this stage of my trouble cause a relapse, doctor?" inquired the patient. "Yes, and a serious one." "Please then, doctor, to remember that important fact in making out your bill."

